# Ablation of parahisian premature ventricular extrasystoles by subtricuspid retrograde approach using inverted catheter technique: back to the anatomy

**DOI:** 10.1002/ccr3.1467

**Published:** 2018-03-08

**Authors:** Basar Candemir, Veysel Duzen, Firat Coskun, Veysel Kutay Vurgun, Huseyin Goksuluk, Nil Ozyuncu, Seda Tan Kurklu, Ali Timucin Altin, Omer Akyurek, Cetin Erol

**Affiliations:** ^1^ Department of Cardiology Ankara University Ankara Turkey; ^2^ Department of Cardiology Gaziantep Ersin Arslan Research Hospital Gaziantep Turkey

**Keywords:** Catheter ablation, premature ventricular contraction, ventricular arrhythmia

## Abstract

This report illustrates a feasible and anatomical solution aiming to improve the success and decrease the possible hazards such as atrioventricular block during ablation of parahisian PVCs. We tried to illustrate the specific anatomy pertaining parahisian region and to explain a retrograde subvalvular catheter technique to overcome these procedural obstacles.

## Introduction

Radiofrequency ablation of PVCs originating from parahisian region can be frequently challenging because they have not only high risk of iatrogenic atrioventricular block, but also have lower long‐term success rates than other locations. In this technical report, we start with a case presentation in which Rf ablation via a large‐loop inverted catheter successfully eliminated parahisian premature ventricular extrasystoles (PVC) focus located under the septal leaflet of tricuspid valve after various antegrade approaches failed and then discuss the relevant anatomy and catheter techniques which could be utilized on PVCs originating from the parahisian region.

While the majority of idiopathic premature ventricular contractions (PVC) arise from right or left ventricular outflow tracts and aortic sinuses of Valsalva, a minority of them originate from other areas including annuli, papillary muscles, and parahisian region 1, 2, 3. Radiofrequency (Rf) ablation of PVCs from parahisian region can be frequently challenging because they not only have high risk of iatrogenic atrioventricular (AV) block, but also have lower long‐term success rate than other locations 1. In this case report, we start with a case presentation in which Rf ablation via a large‐loop inverted catheter successfully eliminated a parahisian PVC focus located under the septal leaflet of tricuspid valve after various antegrade approaches failed and then discuss the relevant anatomy and ablation techniques which could be utilized in similar PVCs.

## Case Presentation

A 67‐year‐old male patient with highly symptomatic PVCs (ambulatory Holter monitor: 31.562 PVCs/112.680 total beat; PVC burden: 28%) was referred to our clinic for catheter ablation. He reportedly underwent a coronary bypass grafting surgery 5 years ago, and his coronary angiography which was performed recently revealed no significant stenosis with completely patent 2 vascular grafts. His echocardiogram was unremarkable with normal biventricular size and function. PVCs were reported to be completely refractory to beta blockers and non‐DHP calcium channel blockers for the last 2 years. His physical examination was again unremarkable except for very frequent extrasystoles. An electrocardiogram (ECG) revealed frequent, mostly bigeminy, monomorphic PVCs with LBBB pattern, monophasic R wave in DI, DII, and aVL, and precordial transition between V2 and V3.

The electrophysiologic study was performed using a electroanatomic mapping system (Carto 3 Mapping System, BioSense Webster, Diamond Bar, CA, USA) and one quadripolar atrial mapping catheter and one irrigated mapping and ablation catheter (ThermoCool, BioSense Webster, Diamond Bar, CA, USA) inside a steerable sheath. Initial activation mapping did not yield a “sufficiently early activation” after the mapping of middle cardiac vein, proximal right ventricular outflow tract (RVOT), right paraseptal, and supravalvular and infravalvular left ventricular outflow tracts (LVOT). The earliest site was located on the tricuspid annulus (−12 msec), adjacent to the His bundle anterosuperiorly with a wide‐area simultaneous activation with a small His potential on the proximal bipole during sinus beats.

**Figure 1:**
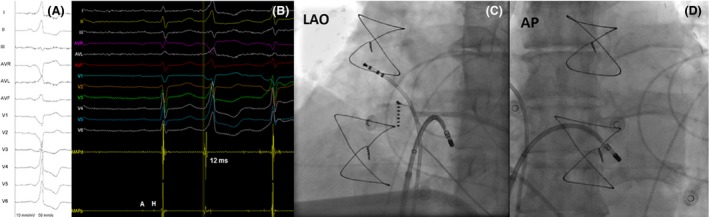
(A) ECG of PVC; (B) local electrogram preceding the QRS by 12 msec at the earliest site during antegrade mapping. Note that the proximal bipole has his and atrial signal in sinus beats. (C and D) left anterior oblique and anteroposterior views of the ablation catheter, respectively.

After informing the patient of potential hazards and risk of complications, several power‐titrated Rf ablations were delivered at right and left parahisian regions (supravalvular and infravalvular), starting at sites away from His potential recording regions with Rf levels starting from 5Watts up to 35 Watts (Fig. 1). During the Rf delivery, the PVCs vanished transiently but consistently persisted later. Afterward, the earliest ventricular sites were targeted avoiding large but small His potentials, but early occurrence of very rapid junctional beats at power levels as low as 5 watts precluded this approach and PVCs persisted.

**Figure 2:**
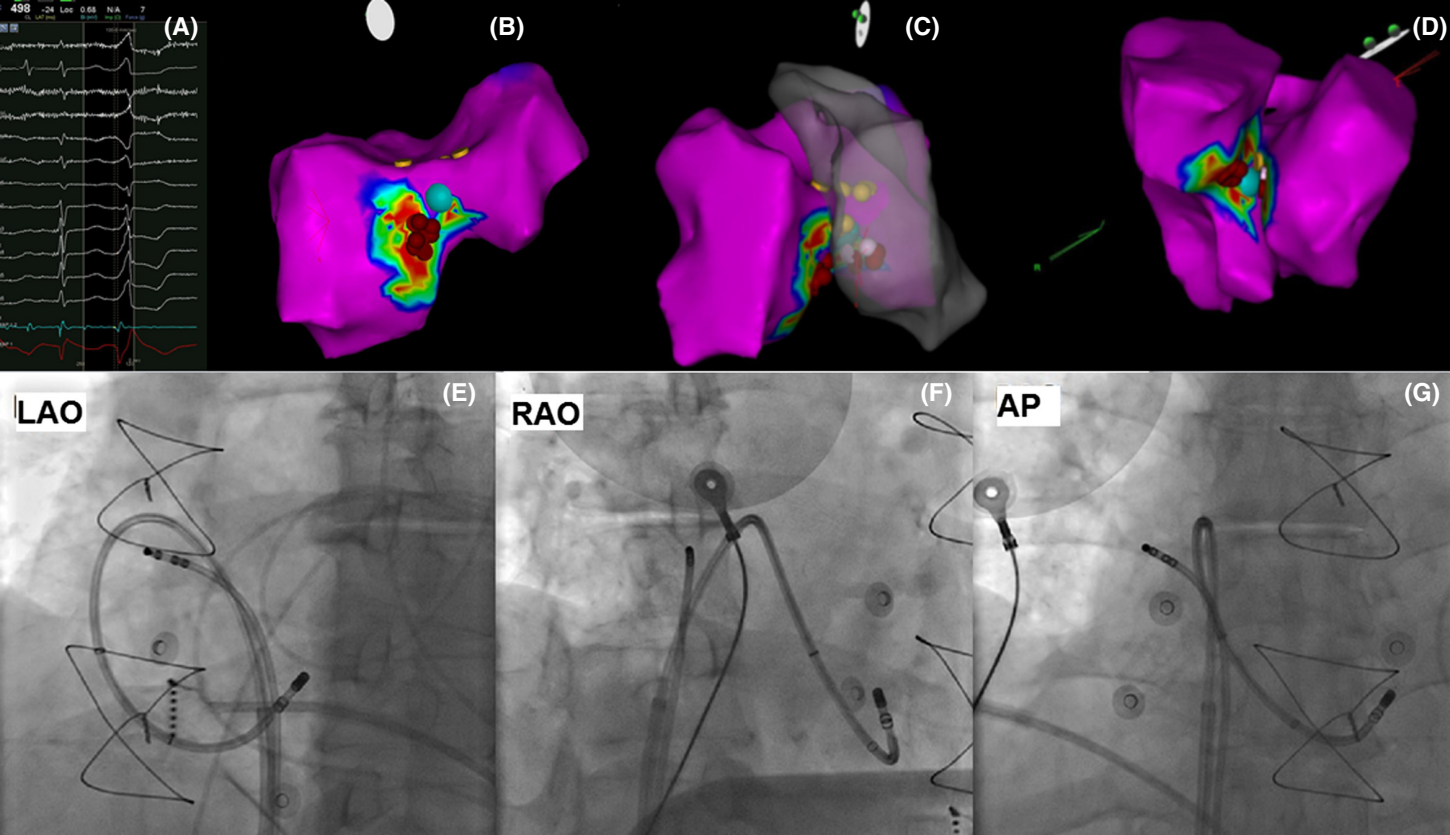
(A) Local electrogram preceding the QRS by 24 msec with a QS signal at the unipolar recording at the retrograde earliest site denoted by the blue dot; (B) right ventricular view of the ventricular septum and ablation lesions; (C) solid right ventricular and transparent left ventricular septum viewed from left anterior oblique projection; (D) left inferior view of confluence of right and left septal walls forming the muscular crest; (E, F, and G) left anterior oblique, anteroposterior, and right anterior oblique projection of the inverted ablation catheter, respectively. *For all electroanatomic maps*, blue dot denotes the earliest and successful ablation site, red and white spots are ablations during antegrade attempt, and yellow spots are where local electrogram has prominent his potentials in sinus beats.

We suspected a subvalvular focus and approached the earliest right parahisian site retrogradely with inverted ablation catheter. The ablation catheter was advanced until it contacted the right ventricular apex. Then, it was progressively flexed while it was advanced under continuous clockwise torque through a steerable sheath held at the level of tricuspid annulus so that its tip finally faced the subvalvular region. We subsequently mapped the subtricuspid region and reached a previously unreachable new site which was actually just under the previously supravalvular “the earliest” region. The local electrogram was preceding the surface QRS by 24 msec and demonstrated QS on unipolar recording without any visible His‐like potential in none of the bipoles (Fig. 2). Again, Rf ablation was delivered to this site starting from 5 W and was cautiously increased to 35Watts for a total ablation time of 3 min (temperature limit 44°C) while monitoring for rapid junctional rhythm. PVCs were eliminated after 5 sec with no junctional rhythm or AV conduction injury. PVC elimination was tested with isoproterenol infusion with rapid pacing and programmed extrastimulation, but no PVCs were observed during the waiting period of 30 min. We also did not observe any atrioventricular conduction disturbance with similar AH and HV intervals. He remained completely asymptomatic afterward, and an ambulatory 24‐h Holter monitor performed after 2 months revealed 28 multiform PVCs.

## Discussion

This report presented a safe and effective catheter ablation technique for challenging parahisian PVCs which constitute 3–9% of all idiopathic PVCs. They can be recognized via specific ECG characteristics such as relatively narrow QRS, LBBB pattern, and slight inferior axis with negative/isoelectric DIII and prominently positive DI, DII, and aVL 1, 2, 3. Despite recent developments in ablation and mapping technologies, neither acute nor long‐term success rate are not ideal still, as acute success rate has been recently reported to be 78% 1. This figure represents both the anatomical and technical difficulties such as poor catheter contact and instability and higher risk of hazards, especially relatively high risk of injury to AV conduction system. Not infrequently, these VAs arise from intramural septal sites which usually require higher Rf energy levels for longer duration compared to a superficial foci.

The HB is a ventricular structure, measuring 20 mm in length and 4 mm in diameter 4, as both inferior compact AV node and HB have been shown to be deriving from ventricular myocardium 5. It is located within the membranous ventricular septum, and after the membranous septum, the HB continues downward as right and left bundle branches over the muscular crest of ventricular septum. Reportedly, It has three different anatomical variations which are as follows: Type 1 (the majority: 47%): The HB runs along the lower border of the membranous septum and is covered with a thin myocardial‐connective tissue insulating layer over the muscular interventricular septum and; Type 2: (32%): The HB runs apart from the lower membranous septum and penetrates and enters the muscular septum; and Type 3: (21%), the HB runs superficially just beneath the endocardial layer and has no overlying myocardial coat while traversing over membranous septum 6. It is also paramount to know that the distal compact AV node and proximal HB have no overlying connective‐muscular tissue layer which practically makes them relatively unprotected to RF injury/AV block 7, 8.

**Figure 3:**
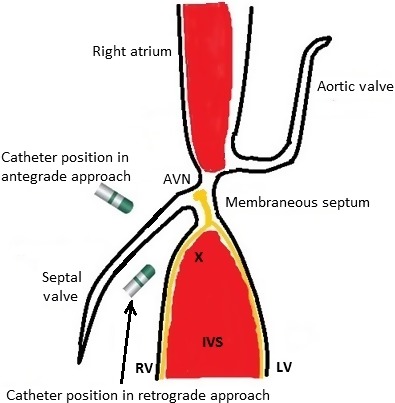
Schematic drawing of parahisian and muscular crest region depicting the anatomical relations and direction of the ablation catheter according to approach route. “X” denotes the hypothetical arrhythmia focus at the muscular crest. His bundle and branches are drawn yellow. AVN, atrioventricular node; IVS, interventricular septum; LV, left ventricle; RV, right ventricle.

The HB and tricuspid valve have also very important relationship as the membranous septum, which has penetrating HB inside, is partly made up from anterior and septal tricuspid commissure on the right side. The septal tricuspid valve completely covers the crest of interventricular muscular septum from where parahisian PVCs are presumably originating (Fig. 3). This specific anatomical region has very important practical implications such that, as we experienced in our case, valvular tissue might act as a heat shield and prevent effective lesion development on the muscular septum. In a possible scenario, even though the activation map leads a physician to a “seemingly” correct, earliest site antegradely, the PVCs typically disappear transiently upon the delivery of Rf and reappear after the energy delivery is stopped. In those cases, longer duration, higher level Rf energy levels over tricuspid septal valvular tissue might be successful on a superficially located focus along with highly impending risk of valvular and AV conduction injury. Another option would be selecting a retrograde approach with an inverted catheter aiming to reach the subvalvular area in order to contact directly with basal muscular septum. Nevertheless, it is still not unlikely to record a relatively small His potential at the earliest site which could be due to far‐field HB or near‐field right bundle potential, but application of Rf energy would be more likely to be successful in eliminating the PVCs with relatively low risk of injury to the part of AV conduction system at this site, that is, well‐protected distal HB with insulating layer.

No matter which approach is taken, RF delivery in this specific anatomical region should always be performed with power titration, starting from low levels and incrementing gradually while carefully monitoring for AV conduction injury, that is, accelerated junctional beats or AV dyssynchrony. We believe that septal atrial signal should be also be monitored during ablation using a separate quadripolar mapping catheter in order to discriminate rapid arrhythmic focus firing due to heating from rapid junctional beats. It is also paramount to avoid ablation over relatively unprotected proximal HB which can be identified as AV electrogram amplitude 1:1–1:2 with visible His potential and absence ventricular potential on proximal bipole 9, 10. Although the cryoablation could be thought as a safe alternative to Rf energy at parahisian location, the utilization and the safety of cryo energy for parahisian PVCs have not been reported yet, to our knowledge.

## Authorship

BC: prepared the manuscript. BC, FG, VKV, OA, and ATA: performed the procedure. VD, HG, NO, and STK: reviewed the manuscript. CE: reviewed the manuscript for syntax and typographical error.

## Conflict of Interest

None.
